# Effect of ghrelin on serum metabolites in Alzheimer’s disease model rats; a metabolomics studies based on 1H-NMR technique

**DOI:** 10.22038/ijbms.2018.30596.7373

**Published:** 2018-12

**Authors:** Fatemeh Goshadrou, Afsaneh Arefi Oskouie, Maryam Eslami, BiBi Fatemeh Nobakht Mothlagh Ghoochani

**Affiliations:** 1 Faculty of Paramedical Sciences, Department of Basic Sciences, Shahid Beheshti University of Medical Sciences, Tehran, Iran; 2 Department of Physiology, School of Medicine, Iran University of Medical Sciences, Tehran, Iran; 3 Department of Basic Medical Sciences, Neyshabur University of Medical Sciences, Neyshabur, Iran

**Keywords:** Alzheimer’s disease, Ghrelin, Metabolic profiling, Metabolomics, Nuclear magnetic resonance

## Abstract

**Objective(s):**

Alzheimer’s disease (AD) is dysfunction of the central nervous system and as a neurodegenerative disease. The objective of this work is to investigate metabolic profiling in the serum of animal models of AD compared to healthy controls and then to peruse the role of ghrelin as a therapeutic approach for the AD.

**Materials and Methods:**

Nuclear magnetic resonance (NMR) technique was used for identification of metabolites that are differentially expressed in the serum of a rat model of the AD with or without ghrelin treatment. Using multivariate statistical analysis, models were built and indicated.

**Results:**

There were significant differences and high predictive power between AD and ghrelin-treated groups. The area under curve (AUC) of receiver operating characteristic (ROC) curve and Q^2^ were 0.870 and 0.759, respectively. A biomarker panel consisting of 14 metabolites was identified to discriminate the AD from the control group. Another panel of 12 serum metabolites was used to differentiate AD models from treated models.

**Conclusion:**

Both panels had good agreements with clinical diagnosis. Analysis of the results displayed that ghrelin improved memory and cognitive abilities. Affected pathways by ghrelin included oxidative stress, and osteoporosis pathways and vascular risk factors.

## Introduction

Alzheimer’s disease (AD) is a multifactorial progressive, neurodegenerative disorder that leads to loss of memory and cognitive deficits. Aging of the brain is strongly appended by morphological and neurophysiological modifications that cause impairments in the learning and memory and modifications in metabolic status (1). There are no reliable diagnostic biomarkers for this disease (2). The main features of AD are the accumulation of amyloid-ß (Aβ) plaques and the tau protein (3). There are some approaches fosr diagnosis of the AD, such as the examination of amyloid levels in the cerebral spinal fluid (CSF) and levels of phosphorylated tau protein (4). Furthermore, imaging techniques such as positron emission tomography (PET) and proton magnetic resonance spectroscopy (MRS) (5, 6) are useful procedures for identifying the plaque load. Despite their advantages, imaging techniques have some limitations such as low sensitivity and working based on only amyloid and tau factors; therefore, the accuracy of early stage prediction by these methods is very limited. In addition, imaging techniques are expensive and their maintenance costs are high. Hence, identification of AD in the early stages of the disease, before Aβ protein aggregation, tau hyperphosphorylation and tangle formation will be useful.

In recent years, researchers have special attention to metabolomic studies, because it allows monitoring the perturbations of homeostasis in the systems biology. Metabolomics has been defined by Nicholson as “quantitative measurement of the dynamic multiparametric metabolic response of living systems to pathophysiological stimuli or genetic modification” (7). Metabolomics is complementary to genomic, transcriptomic and proteomic variations. Metabolites have the closest situation to phenotype because they are emblematic of interactions between genes, proteins, and the environmental effects such as disease, therapeutic interventions, stress, and so on. Metabolomics studies represent an accurate understanding of biochemical pathways and mechanisms of diseases. In fact, one of the important missions of the metabolomics is the introduction of the metabolite biomarkers (8). 

A variety of analytical techniques have been applied in metabolomic studies and screening of the metabolites. The most common analytical instruments that have been employed are nuclear magnetic resonance (NMR) spectroscopy and mass spectrometry such as gas chromatography-mass spectrometry (GC-MS) and liquid chromatography-mass spectrometry (LC-MS) (9). Among the above techniques, NMR is widely used (10, 11) because of its advantages such as fast analysis, being non-destructive and cost-effective. NMR also demands little or no sample preparation and produces repeatable and reproducible results.

Currently, treatments for the AD are based on stabilizing and minimizing disruption of neurotransmitters of acetylcholine (ACh) and glutamate. Acetylcholinesterase (AChE) inhibition is used to protect the cholinergic neurons and glutamate (12). Donepezil, Rivastigmine, and Galantamine are three compounds that have been used based on AChE inhibition. The Memantine is another medication for AD that acts as a blocker of N-Methyl-D-Aspartate (NMDA) receptors. However, the efficacy of these drugs is low (13, 14). Therefore, the search for therapeutic agents in all phases of the AD is required. Till now, there is no single drug that stops or reverses the pathological process of this chronic disease completely. 

Ghrelin as a peptide-based ligand (composed of 28-amino acid), is the growth hormone secretagogue (GHS) receptor and increases the appetite (15, 16). Obesity and the metabolic syndrome are directly related to dysregulation of ghrelin (17, 18). Ghrelin plays an important role in inflammation (19) and neuromodulation (20, 21). Biomarkers are extremely useful in the assessment of the efficacy of drugs and scrutiny of novel therapeutic areas, which have not been previously studied. In other words, biomarkers are the bridge between candidate drugs and clinical effectiveness of these drugs. Therefore, utilization of metabolomics technologies in biomarker discovery has attracted much attentions and was one of our goals in this study (22).

A literature review showed that most of the studies used CSF for diagnosis of AD. The composition of this biofluid is directly reflection of the metabolite alterations in the brain, but the acquisition of CSF is invasive. Previous studies indicate that the metabolomic studies in AD disease have attracted much attention from research teams by a variety of techniques such as NMR, GC- MS and LC-MS (23). 

Despite the numerous studies that have been performed in this area, the mechanism of the disease is still poorly understood. In this paper, we performed a metabolic analysis of Aβ - induced neuroinflammation in AD rat models and co-treatment with the peptide-based compound, ghrelin in a AD model rats. All the spectra were analyzed using multivariate statistical algorithms to determine the integrity of metabolites that change with disease progression. 

## Materials and Methods

***Animals***

The procedures in this section were performed according to our previous studies (24). Forty adult male albino Wistar rats (Pasteur Institute, Tehran, Iran) weighing 250 ±20 g were housed in cages of four in a temperature-controlled holding room (23 ± 1^o^C) on a 12/12 hr light/dark cycle (lights on at 7:00 am). Standard laboratory, food, and water were available *ad libitum* throughout the study. Animals were allowed to become accustomed to the environment and were handled for a week prior to the tests. Also, efforts were made to minimize animal discomfort and reduce the sample size. All experiments were performed during the light phase and in accordance with the Guide for the Care and Use of Laboratory Animals (National Institute of Health Publication No. 80-23, revised 1996) and were confirmed by the Research and Ethics Committee of Shahid Beheshti University of Medical Sciences.

In the current study, animals were randomly assigned to 5 different groups :a) Intact or control group (n=8) was subjected to neither stereotaxic surgery nor drug administration during the study. b) Sham-operated (Sal+Sal) group (n=8) was administered a single microinfusion of 5 µl of saline (Sal; as Aβ1-42 vehicle) into the lateral ventricles (LV), immediately after the cannulation surgery. After a week of recovery, this group received 2 µl of saline (as Aβ1-42 or ghrelin vehicle) into the LV for 2 weeks. c) Sal + Ghr (ghrelin-control) group (n=8) also received a single microinfusion of 5 µl of saline (as ghrelin vehicle) into the LV, immediately following the stereotaxic surgery. After a week of recovery, this group was microinjected with ghrelin at the dose of 200 ng/rat for 2 weeks. d) Aβ + Sal group (n=8) was administered a single microinfusion of 5 µl of Aβ1-42 into the LV, immediately after the stereotaxic surgery. After a week of recovery, this group received 2 µl of saline (as ghrelin vehicle) into the LV for 2 weeks. e) Aβ + Ghr group (n=8) received a single microinjection of 5 µl of Aβ1-42 into the LV, immediately following the cannulation surgery. After a week of recovery, this group was microinjected into the LV with ghrelin at the dose of 200 ng/rat for 2 weeks. Animals in sham-operated, Sal + Ghr, Aβ + Sal and Aβ + Ghr groups underwent the behavioral studies on the following day of the last microinjection. Blood sampling was performed at the end of behavioral experiments. 

***Drugs***

An animal model of the AD was created by intracerebroventricular (ICV) injection of Aβ1-42. Aβ-peptide (1-42) (Tocris Bioscience, Bristol, UK) was dissolved in sterile normal saline solution (0.9% NaCl) at a concentration of 1 μg/μl and incubated at 37 ^°^C for 7 days to induce aggregation. Acylated ghrelin (Sigma-Aldrich, St. Louis, USA) as a ligand for G-protein coupled receptor (GPCR), and growth hormone secretagogue receptor (GHS-R1A) were also prepared in saline at 1 μg/μl, divided into aliquots and stored at -20 ^°^C until use. Administration of ghrelin was performed at the dose of 200 ng/rat (5 µl, 1 nM). This dose of ghrelin was chosen based on our pervious study. All peptides were dissolved and prepared for the experiments.

***Stereotaxic surgery and drug administration***

Rats were intraperitoneally (IP) anesthetized by a solution of xylazine 2% and ketamine 10% (15 and 85 mg/kg, respectively; Alfasan, Woerden-Holland) and mounted on a stereotaxic apparatus (Stoelting, USA). After the scalp was incised and the skull was cleaned, a guide cannula (23 gauge, 9 mm) was unilaterally implanted 1 mm above the lateral ventricle, using stereotaxic coordinates (AP = -1.2 ± 0.2 mm; ML = 1.5 ± 0.5 mm; DV = -4 mm) from the rat brain atlas (Paxinos and Watson, 2007). Because it is necessary to fix the cannula in place firmly, we used two stainless steel screws anchored to the skull and dental acrylic cement. The cement should be completely dry and hardened; afterwards, a dummy cannula (30 gauge) was inserted into each guide cannula to prevent clogging and infection. Rats were housed individually and allowed to recover for a week before the experiments.  

ICV microinfusions of 5 μl of saline, Aβ1-42 or ghrelin solutions were separately carried out over 2 min during the experiments, using a 5 μl Hamilton syringe connected to a 10 mm microinjector (30 gauge) through a piece of polyethylene tubing (PE-20). In order to minimize backflow along cannula tract and to facilitate diffusion of the drugs, the injector was left in situ for an additional 1 min each time before it was slowly withdrawn. Animal stress was reduced by moving freely during the infusion procedure. 

***Passive avoidance task***

The shuttle box apparatus was used to study and evaluate passive avoidance memory with slight modifications to efficiently examine the memory performance during habituation session in addition to the recovery. It was divided by a guillotine door into two equal sized light and dark compartments (20 × 20 × 40 cm). The floor of each of the chambers was covered with stainless steel rods spaced 1 cm apart and only the grid floor in the dark chamber was connected to a shock source. Before the training trials, animals were habituated to the apparatus by placing them in the light chamber. After 5 sec, the door was removed to allow the rats free access between both compartments for 10 min, while just five intermittent foot shocks of 0.5 mA for 1 sec (to avoid any stress) was applied in the dark chamber. Following the first habituation, animals were put in the light chamber and again after 5 sec the sliding door was removed, but as soon as the animals enter the dark chamber the door was lowered and the rats were returned to their cages after 20 sec without receiving any foot shock. Animals that did not enter the dark chamber within 30 sec were excluded from the study. The habituation trial was repeated after 30 min. The entrance latency to the dark compartment (step through latency, STL) was recorded for each animal (25, 26). The training trial was performed 30 min after the second habituation session. During the training, animals received a foot shock (a 1.2 mA, 50 Hz current for 1.5 sec) immediately after they crossed to the dark chamber. Rats were left in the dark compartment for 20 sec before they were temporarily returned to their cages. After 2 min, the rats were again placed in the light chamber to see whether they stayed there for 2 min or not. It was considered as a successful learning when the animals did not enter the dark chamber during that period, otherwise they would receive the same electric shock again until they refrain from entering the dark compartment. Twenty four hours later, to examine the memory retrieval, the rats were again put in the light chamber for 5 sec before removing of the guillotine door, while no foot shock was applied in this trial. STL, time spent in the light and dark compartments (TLC and TDC, respectively) and the number of crossings between the two chambers were then recorded for 10 min.

***Blood sampling***

Blood sampling was performed at the end of behavioral experiments. After centrifuging the blood samples at 3000 rpm at 4 ^°^C for 10 min, the collected serum was stored at − 80 ^°^C until applied for NMR analysis.

***NMR acquisition***

The NMR approach was applied similar to our previous studies (27, 28). The NMR spectra were acquired using a Bruker DRX500 MHz spectrometer operating at 500.13 MHz, equipped with 5 mm high-quality NMR tubes (Sigma-Aldrich, RSA). Serums were thawed at room temperature, and 250 μl from each sample was mixed with 10% D_2_O (deuterium oxide, 99.9% D, Aldrich Chemicals Company) in order to lock the signals. The chemical shifts of the samples were referenced to DSS (3-(Trimethylsilyl) -1-propane sulfonic acid-d6 sodium salt, 98% D, Sigma- Aldrich), as an internal standard. The ^1^H-NMR spectra of serum samples were recorded at a constant temperature of 298 K (25 °C) using the Carr–Purcell–Meiboom–Gill (CPMG) spin-echo pulse sequence, π/2-t_D_-π-t_D_, to facilitate the detection of low molecular weight species (29, 30). The acquired ^1^H NMR spectra were recorded with 128 scans, 32 dummy scans, and a spectral width of 8389.26 Hz, with an acquisition time of 1.95 sec, and a relaxation delay of 2 sec.

***NMR data processing***

All raw data or free induction decay (FID) were corrected for phase and baseline using the XWINNMR (version3.5, Bruker Spectrospin Ltd) manually. The chemical shifts of δ 0.2–10 ppm were subdivided into integrated regions of 0.02 ppm width (bin) using the ProMetab software (version prometab_ v3_3) (31, 32) in MATLAB (Version 6.5.1, The MathWorks, Cambridge, UK). The region of δ 4.5 to δ 5.5 ppm that contained the residual water signal was excluded. One-dimensional ^1^H NMR data were normalized before pattern recognition analysis and aligned using ProMetab software in the MATLAB software. Each bin was normalized using total spectral area, log-transformed, and mean-centered before pattern recognition analysis. Then, the final data were imported to SIMCA version 14.0 (Umetrics, Umea, Sweden) for multivariate analysis. 

***Statistical analysis***

SIMCA (SIMCA 14.0, Umetrics, Umeå, Sweden) and SPSS 16.0 (SPSS, Inc., Chicago, IL) were used for all analyses. First, principal component analysis (PCA) was utilized to the spectral data to find any intrinsic clustering and find obvious outliers within the data set. Then, the data were subjected to orthogonal projections for latent structures-discriminant analysis (OPLS-DA). This approach is used to construct predictive models and identify metabolite fingerprint differences. R^2^ and Q^2^ are important parameters and usually apply for evaluation of the performance of the models. R^2^ and Q^2^ are respectively the class separator and predictive power of the model (33, 34). The ranges of these parameters are between 0 and 1. Furthermore, receiver operating characteristic (ROC) curve was applied to evaluate the OPLS-DA. Performance prediction and the area under the ROC curve (AUC) value was reported with the 95% confidence interval. 

***Metabolite identification and metabolic pathway analysis***

The highest importance spectral bins were selected and the *P*-value and fold change were calculated for each bin. Those bins with *P*-values of less than 0.05 and the fold change of more than 1.5 were brought to databases for identification. Significantly altered metabolites were found with the matching of the selected NMR spectral bins with the available data in the metabolomics databases including human metabolome database (HMDB), with an accuracy of ±0.03 ppm. Pathway analysis was performed to introduce affected metabolic pathways in AD pathogenesis using the web server, Metaboanalyst (http://www.metaboanalyst.ca/) (35-37). Pathway analysis combines pathway enrichment analysis for *Rattus norvegicus*, hypergeometric overrepresentation test, and topology analysis.

## Results

***Behavioral tests***

AD approval of the animals was performed by the behavioral test. Step-through passive avoidance task was accomplished as described previously. Behavioral test results are shown in Table 1. Our results showed that no significant difference was observed between five groups [F (4,41): 0.5019, P=0.750] in the STL. This also accords with our earlier observations, which showed that similar STL of rats before the acquisition trial are proof of the behavioral homogeneity of the animals [37, 38]. However, after the training, in the retrieval trial STL in ghrelin group (Sal+Ghr) was significantly higher than the controls (intact) group (*P*<0.001). The result of Tukey’s *post hoc* test revealed that the STL time of Alzheimer’s model group (Aβ+Sal) was shorter than control (intact) and ghrelin (Sal+Ghr) group (*P*<0.05). Injection of ghrelin (ICV) in a rat model of AD relatively recovers rats to the intact group in this task and there was no difference between intact and Aβ+Sal groups. 

**Figure 1:**
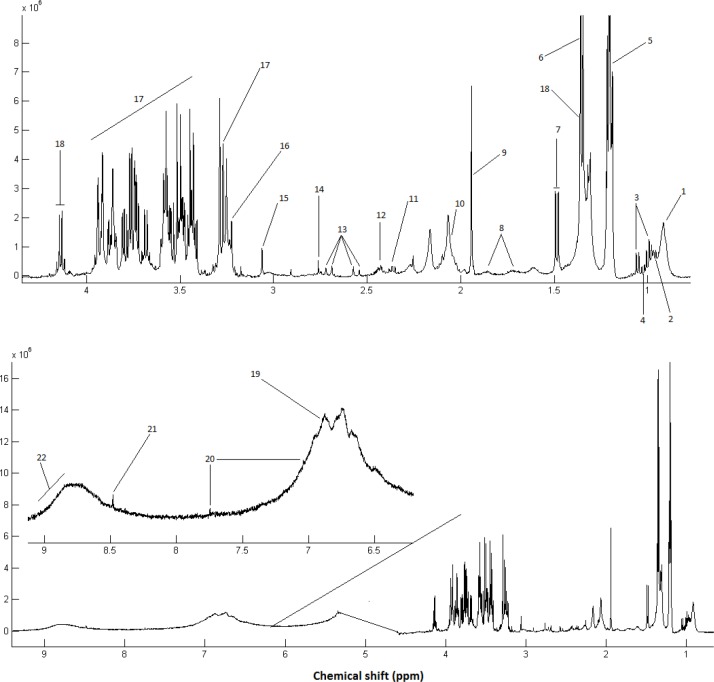
Representative 500 MHz one-dimensional Carr–Purcell–Meiboom–Gill (CPMG) ^1^HNMR spectrum of rats with Alzheimer’s disease (AD) treated with ghrelin; serum samples measured at 298 K. The following metabolites were identified: 1, Lipid (mainly LDL and VLDL), 2, Leucine; 3, Valine; 4, Isoleucine; 5, 3-Hydroxybutyrate; 6, Threonine; 7, Alanine; 8, Lysine; 9, Acetate; 10, Lipid: CH2-CH=CH ; 11: Glutamate; 12, Glutamine; 13, Citrate; 14, Lipid: C=CCH2C=C; 15, Creatine; 16, Choline; 17, α & β-Glucose; 18, Lactate; 19, Tyrosine; 20, Histidine; 21, Formate; 22, 8-Hydroxyguanosine

**Figure 2:**
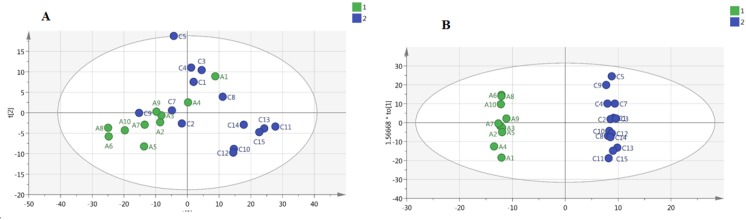
a) Principal component analysis (PCA) model between Alzheimer’s disease (AD) and control group. b) Orthogonal projections to latent structures-discriminant analysis (OPLS-DA) model between AD and control group

**Figure 3:**
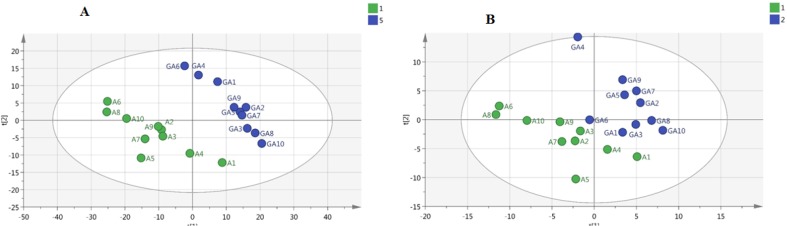
a) Principal component analysis (PCA) model between Alzheimer’s disease (AD) and treated group. b) Orthogonal projections to latent structures-discriminant analysis (OPLS-DA) model between AD and treated groups

**Figure 4.**
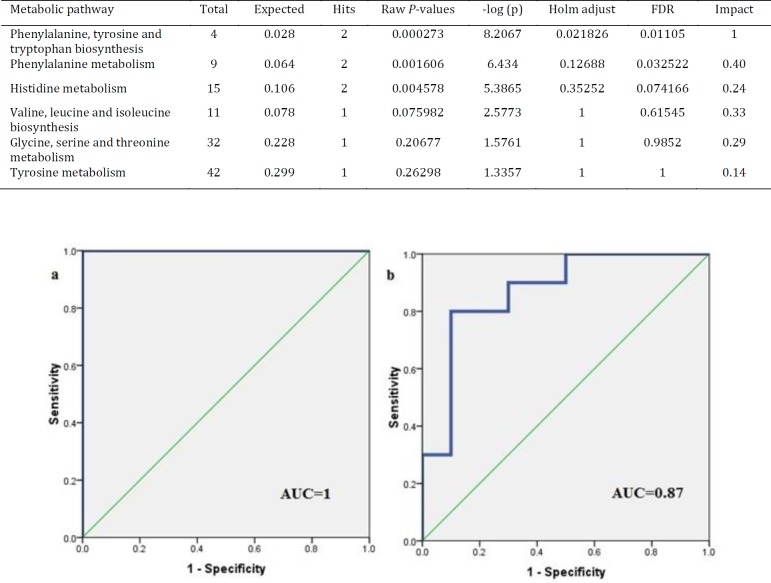
Validation of multivariate models of NMR data. (a) Receiver operating characteristic (ROC) curve analysis of the predictive power of serum biomarkers for distinguishing Alzheimer’s disease (AD) from controls using orthogonal projections to latent structures-discriminant analysis (OPLS-DA model). (b) ROC curve analysis for the predictive power of serum biomarkers for distinguishing AD from the treated - group using OPLS-DA

**Figure 5:**
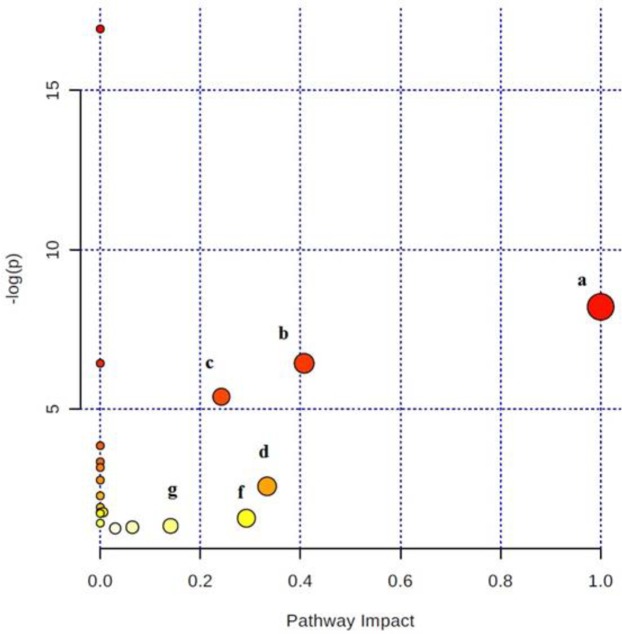
Plot of the results of pathway analysis in Metaboanalyst for the Alzheimer’s disease (AD) group after treatment with ghrelin. Those pathways with *P*-values less than 0.05 are ranked according to their impacts: a: Phenylalanine, tyrosine and tryptophan biosynthesis, b: Phenylalanine metabolism, c: Histidine metabolism,d: Valine, leucine and isoleucine biosynthesis, f: Glycine, serine and threonine metabolism, g: Tyrosine metabolism

**Table 1:** Step-through latency (STL) as a memory index in different groups before and after acquisition trials. Results are expressed as means±SEM

**Groups**	**Control** **(Intact)**	**Sal+Sal**	**Sal+Ghr**	**β-amyloid+Sal**	**β-amyloid+Ghr**
**Before acquisition trial **(sec)	10.65 ±0.95	10.30±0.73	8.70±0.45	13.06±0.06	11.37±0.69
**After acquisition trial **(sec)	156±60.32	199±61.10	493.70±72.70	30.25±4.02	109.125±11.50

**Table 2:** Differential serum metabolites between Alzheimer’s disease (AD) and control group using NMR

**No.**	**Metabolite name**	**Observed Chemical shifts(ppm)**	**Fold change**	**Direction**
**1**	Cholesterol	0.7, 0.8	2.26	
**2**	Stearic Acid	1.01,1.44,1.77, 2.48	1.6	
**3**	Alanine	1.47, 3.76	10.46	
**4**	Valine	0.97, 1.02, 2.26, 3.59	2.78	
**5**	Lysine	1.71, 1.89,3.03, 3.76	10.46	
**6**	2-Oxoglutarate	1.87, 2.3, 3.0	2.93	
**7**	Acetate	1.91	1.69	
**8**	Glutamate	2.46	4.60	
**9**	Creatine	3.03,3.92	2.36	
**10**	Homocysteine	2.14, 2.65, 3.87	2.1	
**11**	Citrulline	1.56, 1.87, 3.14,3.74	2.19	
**12**	Aspargine	2.85, 2.95, 4.0	1.76	
**13**	Glucose	3.23, 3.39, 3.45,3.52, 3.72, 3.82, 3.88, 4.63, 5.23	2.29	
**14**	Acetoacetate	2.23, 3.43	1.56	

**Table 3:** Altered metabolites after treatment of Alzheimer’s disease (AD) rats with ghrelin compared to AD group

**No.**	**Metabolite name**	**Chemical Shift (ppm)**	**Fold change**	**Direction**
**1**	Homocysteine	2.14, 2.85, 3.87	1.5	**↓**
**2**	Lysine	1.71, 1.89, 3.02,3.74	2.15	**↓**
**3**	Lactate	1.33, 4.11	2.15	**↓**
**4**	Glycine	3.54	1.8	↓
**5**	Valine	0.97, 1.02, 2.24, 3.57	2.27	↓
**6**	Proline	1.99, 2.05,3.34,3.45	1.6	
**7**	Tyrosine	3.06,6.87	1.9	
**8**	1-methyl Histidine	7.05	1.67	
**9**	8-Hydroxyguanosine	8.9-9.2(broad)	6.2	
**10**	Histidine	7.02, 7.74	1.89	
**11**	3-Methylhistidine	7.61	2.0	
**12**	Phenylalanine	3.26,3.97,7.33,7.38,7.43	1.8	

a Increased or decreased in the treated group compared to AD serum.

**Table4.** Results of pathway analysis using MetaboAnalyst 3 for the Alzheimer’s disease (AD) group after treatment with ghrelin. Hypergeometric test is used for the over-representation analysis. The ‘Total’ column shows the number of compounds in each pathway. The ‘Hit’ column is the number of our query metabolites. The raw *P*-values are adjusted with Holm and FDR methods. The ‘Impact’ refers to the impact of each pathway from topological analyses, here, according to the relative betweenness centrality, for measuring the importance of pathways.

**Metabolic pathway**	Total	Expected	Hits	Raw *P*-values	-log (p)	Holm adjust	FDR	Impact
Phenylalanine, tyrosine and tryptophan biosynthesis	4	0.028	2	0.000273	8.2067	0.021826	0.01105	1
Phenylalanine metabolism	9	0.064	2	0.001606	6.434	0.12688	0.032522	0.40
Histidine metabolism	15	0.106	2	0.004578	5.3865	0.35252	0.074166	0.24
Valine, leucine and isoleucine biosynthesis	11	0.078	1	0.075982	2.5773	1	0.61545	0.33
Glycine, serine and threonine metabolism	32	0.228	1	0.20677	1.5761	1	0.9852	0.29
Tyrosine metabolism	42	0.299	1	0.26298	1.3357	1	1	0.14

***Discrimination between Alzheimer’s model, controls and ghrelin drug-treated groups by NMR technique***

A CPMG spectrum of the serum sample from the rats with the AD is shown in Figure 1. Processing of the NMR spectra was performed by the ProMetab software. The result was a matrix including spectral bins corresponding to different metabolites and the observations. 

Classification of the data containing 40 samples was performed using unsupervised methods. At first, PCA was performed to detect intrinsic clustering and possible outliers. No clear separation was observed among rats with AD and controls by PCA. Moreover, no outliers were observed in the score plot. The PCA results are illustrated in Figure 2a: (R^2^=0.867, Q^2^ =0.746). OPLS-DA was then performed for better separation of metabolic patterns and also detection of potential biomarkers between two groups (AD versus control). Predicted response values were: R^2^X=0.874, R^2^Y=0.997, Q^2^=0.953 (Figure 2b). ROC curve was plotted, and its corresponding AUC was 1.0 (Figure 4a).

We also performed a PCA on AD and treated AD groups. Figure 3a shows a PCA of these samples demonstrating clear separation between two groups even within the unsupervised model (R^2^X=0.808, Q^2^=0.666). Figure 3b shows the corresponding OPLS-DA model, reinforcing the separation in metabolite profile between these two groups (Alzheimer’s model and treated AD models) (R^2^X=0.917, R^2^Y=0.948, Q^2^=0.759), and suggesting that the OPLS-DA model was robust. ROC curves were plotted, and AUC were 1.0 (Figure 4a) in distinguishing Alzheimer’s disease (AD) from controls and 0.87 in second study (AD from the treated – group) (Figure 4b).

Our results revealed fourteen different metabolites between controls and rats with AD. Furthermore, 12 different metabolites were identified between AD and AD animals treated with ghrelin. Table 3 and Table 4 show the panels of metabolites that were responsible for the separation of Alzheimer’s model from control and treated AD groups, respectively. 

***Metabolite identification***

Using HMDB database, candidate biomarkers were identified and were introduced based on mached chemical shifts.

As it is observed in Table 2, the levels of some metabolites increased in serum of Alzheimer’s model compared to control group, such as cholesterol, lipid (Stearic Acid), lysine, alanine, valine, acetate, acetoacetate, creatine, α-keto glutarate, homocysteine, and citrulline, while the levels of several others metabolites such as glucose, glutamate, and aspargine were decreased. Furthermore, a number of metabolites were different between AD treated with ghrelin group and AD group. The results are summarized in Table 3. In contrast to the results obtained in the Alzheimer’s group, different results and almost reverse trends were obtained in the treated group. Decreased metabolites were homocysteine, lysine, lactate, glycine, valine, proline, 1-methyl histidine, 8-Hydroxyguanosine, histidine, 3-Methylhistidine, and phenylalanine, while the only increased metabolite was tyrosine. 

***Metabolic pathway analysis***

To elucidate the mechanisms of action of ghrelin drug on the rat models of the AD, biochemical pathways were investigated using Metaboanalyst 3 on the server (35-37), which combines the results of the pathway enrichment analysis and pathway topology for finding the most relevant metabolic pathways. The results are shown in Figure 5. The most significant pathways with the *P*-value less than 0.05 were ranked according to their impact values. Accordingly, the most important pathways in order of importance included phenylalanine, tyrosine and tryptophan biosynthesis, phenylalanine metabolism, histidine metabolism, valine, leucine, and isoleucine biosynthesis, glycine, serine and threonine metabolism, and tyrosine metabolism (Table 4).

## Discussion

Recent evidences and the results of our study showed that Aβ significantly impairs memory and injection of ghrelin rescues certain cognitive impairments induced by Aβ (38, 39). Metabolite analysis of biological specimens could help discover the potential biomarkers associated with the disease and also may provide a better understanding of disease pathogenesis. In this study, using NMR-based metabolomics, we demonstrated that the serum of animal models of AD has a distinct metabolic pattern compared to control group and the AD group treated with ghrelin. These alterations are in specific pathways related to lipid, amino acid and energy metabolism. As far, this is the first study to examine the effects of ghrelin on metabolic profiling in AD. 

Ghrelin is known as a part of the interface regulator between energy metabolism, as well as neuroendocrine and neurodegenerative processes. Ghrelin also takes part in lipid and glucose metabolism (40). The influence of ghrelin on mitochondrial respiration has been approved (41). This makes it an attractive target biomarker and drug candidate for prevention or treatment of neurological disorders.

The present study confirms previous findings and contributes additional evidence; in this study, we found increased levels of stearic acid in the AD model. Stearic acid is one of the saturated free fatty acids (FFAs). Researchers in their studies have shown hyperphosphorylation of the tau protein by fatty acids, which leads to neuronal degeneration through astroglia-mediated oxidative stress (42). It has been demonstrated that hypercholesterolemia is one of the most important risk factors for the AD, and high level of LDL- cholesterol is associated with an increase of amyloid plaque deposits in the brain (43). These findings are in agreement with our results that showed increased levels of FFAs and cholesterol in the AD group in comparison with the control group.

 Some studies described the altered mitochondrial function and cellular respiration in the low oxygen consumption (44, 45). Also, in aging, dysfunctions of the mitochondrial oxidative phosphorylation pathway (OXPHOS) have been demonstrated by Ojami and co-workers (46). They showed that the defect of OXPHOS leads to perturbation of the Krebs Cycle (47). Recently Paglia and co-workers showed dysregulation of mitochondrial aspartate metabolism in the AD by analysis of frontal cortex samples with ultra-high performance liquid chromatography coupled with mass spectrometry (UPLC−HILIC−MS) technique (48, 49). Moreover, other metabolites such as asparagine and creatine are related to impaired mitochondrial function. Creatine is a key component in providing energy from mitochondria to the cytosol (50).

Furthermore, we found decreased levels of glucose in the AD model. The brain for signaling processes demands extensive amounts of energy that it supplied by oxidation of glucose and its products (51). Low glucose metabolism coupled with mitochondrial dysfunction was introduced as one of the main and early indicators of the AD (52, 53). Glucose is the main source of energy for neurons. In the lack of enough glucose, its oxidative products in the mitochondria are alternatives to produce ATP (54). When glucose is low, the other substrates to supply glucose are lactate and ketone bodies (55, 56). Moreover, the levels of lactate, acetoacetate, and acetate are elevated because of mitochondrial dysfunction in the brain, and the metabolic pathways shift from aerobic respiration to glycolytic metabolism. Additionally, elevated concentrations of alanine in serum that is related to pyruvate metabolism is a result of the decreased activity of pyruvate dehydrogenase complex (57, 58). These are consistent with our findings. 

Our data showed increases in valine, lysine and homocysteine levels in AD cases. Valine is an essential amino acid that involves in protein biosynthesis and main biological functions such as mood, immune function, stress response, sleep, and appetite regulation. High levels of lysine, and homocysteine in the AD group reversed in the treated group. Furthermore, high circulating level of homocysteine has been introduced as a common risk factor for the development of AD and cardiovascular disease (59). Some researchers believed that raised homocysteine is associated with damage to the arteries, and homocysteine is thought to cause damage by using oxygen of cells and producing free radicals (60). In the treated AD group, we found decreased levels of homocysteine, which means the reduced rate of oxidative stress by ghrelin. 

Oxidative stress is one of the most important concerned pathways in AD (61). As mentioned previously, ghrelin has multiple functions in the CNS such as neuroprotection and neurogensis. Ghrelin also reduces oxidative stress and improves the antioxidant effect on blood and brain (62). Our results showed that the serum concentration of 8-Hydroxyguanosine, as a marker of oxidative DNA damage, was higher in rats with AD compared to controls. However, the levels of this metabolite were decreased in the treated AD group. These findings confirm that ghrelin inhibits oxidative stress pathway.

Carnosine (composed of β-alanyl-L-histidine) and anserine (composed of β-alanyl-N-methylhistidine) are important metabolites in the brain, muscle, and other excitable tissues. Carnosine acts as an antioxidant and copper chelator, and it is also shown to be participated in the neuroprotective actions and human neurodegenerative disease (63). The carnosinase enzyme splits anserine or carnosine into ß-alanine and 1-methyl-histidine or histidine, respectively. In the serum of patients with Parkinson’s disease, multiple sclerosis and cerebrovascular disease, reduced activity of carnosinase was also found (64). In our study, we found decreases in levels of histidine and its derivative (1-methyl-histidine) that can suggest the reduced activity of carnosinase by ghrelin in the treated AD group. 

As shown in Table 3, the levels of 3-methyl-histidine in the treated group were decreased. Literature survey shows that the concentration of 3-methyl-histidine can be an index of the protein degradation rates in muscle, and ghrelin inhibits muscle protein breakdown in rats (65).

Tyrosine is a neurotransmitter precursor of L-dopa, dopamine, norepinephrine, and epinephrine, which was lower in the AD compared to the treated AD group. Furthermore, in the present study, higher serum phenylalanine concentration in the AD in comparison with the treated AD group was reported. Since phenylalanine is the precursor of tyrosine, presumably conversion of phenylalanine to tyrosine as well as the biosynthesis of dopamine, norepinephrine and epinephrine neurotransmitters can be affected. In agreement with our results, Gazit and co-workers showed that accumulation of phenylalanine leads to the formation of fibrils with amyloid-like morphology through the self-assembled mechanism in the hippocampus of mice models and causes the production of Alzheimer’s Aβ (66-68).

 In the present study, a robust set of biomarkers for detecting rats with the AD was established. These findings suggest that metabolomic-based studies not only can distinguish AD group compared to healthy controls, but also can help to find effective treatments for the AD. 

Our study also had a few limitations that are worth mentioning. First, a small sample size of rats in each group. Second, presented diagnostic biomarkers still need confirmation through additional metabolomics techniques.

## Conclusion

A metabolomic study based on NMR technique was employed for the study of AD pathogenesis and treatment with ghrelin, a peptide- based compound. This study, enabled us to compare the serum metabolic profiling of an animal model of AD treated with ghrelin. Our results showed that the treated AD rats had improvements in memory and cognitive abilities as it was shown by passive avoidance task. We found changes in the metabolic profile of two groups. The main metabolites and key pathways involved were identified. These metabolites mostly involved in energy, lipid, and amino acid metabolisms. This is the first study of assessing metabolomics with the treatment of ghrelin and can lead to better diagnosis and understanding the detailed molecular mechanism of disease by a non-invasive method. Our research showed that ghrelin has effects in pathways such as oxidative stress, vascular risk factors and partly compensates some of the failed paths. However, future studies on the current topic with more focus on ghrelin is therefore suggested. 

## Conflicts of Interest

The authors report no conflict of interest. The authors alone are responsible for the content and writing of this article.
